# Psychic and Cognitive Impacts of Cardiovascular Disease: Evidence from an Observational Study and Comparison by a Systematic Literature Review

**DOI:** 10.3390/medsci13030105

**Published:** 2025-08-01

**Authors:** Irene Cappadona, Anna Anselmo, Davide Cardile, Giuseppe Micali, Fabio Mauro Giambò, Francesco Speciale, Daniela Costanzo, Piercataldo D’Aleo, Antonio Duca, Alessia Bramanti, Marina Garofano, Placido Bramanti, Francesco Corallo, Maria Pagano

**Affiliations:** 1 IRCCS Centro Neurolesi Bonino-Pulejo, Via Palermo, S.S. 113, C.da Casazza, 98124 Messina, Italy; irene.cappadona@irccsme.it (I.C.);; 2 Department of Medicine, Surgery and Dentistry, University of Salerno, 84081 Baronissi, Italy; 3 Faculty of Psychology, Università degli Studi eCampus, Via Isimbardi 10, 22060 Novedrate, Italy

**Keywords:** Cardiovascular disease, neuropsychology, depression, anxiety, neurorehabilitation, psychological support

## Abstract

**Background/Objectives**: Cardiovascular diseases (CVDs) are frequently associated with psychiatric and cognitive comorbidities. These conditions have been shown to significantly impact quality of life and clinical outcomes. This study aims to evaluate the prevalence of anxiety, depression, and cognitive deficits in patients with CVD and to compare the results with existing evidence in the literature. **Methods**: A total of 74 patients were assessed using the following standardized screening tools: Montreal Cognitive Assessment (MoCA), Mini-Mental State Examination (MMSE), Beck Depression Inventory-II (BDI-II), and Beck Anxiety Inventory (BAI). A systematic review was then conducted to compare the findings with those reported in the literature. **Results**: Most previous studies using the MoCA reported an over 70% absence of cognitive impairment, whereas this study shows a balanced distribution between the absence of (32.4%) and mild (35%) or moderate (32%) impairment. Studies with the MMSE indicated high rates of absence of cognitive deficits (74–79%), but here, the rate of absence was lower (58%), with an increase in mild impairment (42%). Regarding depression, compared with studies showing only absence or moderate/severe forms, this study reveals a more balanced profile, with 57% without depression and with varying severity levels (22% mild, 19% moderate, and 3% severe). Finally, for anxiety, unlike previous asymmetric distributions, greater variability was observed, with 58% without anxiety and significant percentages of mild (26%), moderate (12%), and severe (4%) anxiety. **Conclusions**: The results highlight a significant and varied prevalence of anxiety, depression, and cognitive deficits, emphasizing the importance of a multidimensional assessment to improve clinical management and therapeutic outcomes.

## 1. Introduction

Cardiovascular diseases (CVDs) represent one of the leading causes of morbidity and mortality worldwide, accounting for approximately 17.9 million deaths each year [1,2]. This group of conditions includes coronary artery disease, cerebrovascular disease, rheumatic heart disease, heart failure, and arrhythmias [2]. Between 1990 and 2019, the global incidence of CVDs nearly doubled—from 271 million to over 523 million cases—while related deaths increased from 12.1 to 18.6 million during the same period [3]. The World Health Organization estimates that annual deaths from CVDs could exceed 23 million by 2030, driven by population aging and behavioral risk factors such as smoking, physical inactivity, and an unhealthy diet [2,3].

In addition to biological and behavioral factors, increasing attention is being paid to the role of mental health in CVD. Conditions such as depression and anxiety not only impair quality of life but also negatively affect prognosis, increasing the risk of cardiac events and mortality. This trend is influenced by several factors, including atherosclerosis and vascular inflammation, which represent crucial biological mechanisms linking cardiovascular diseases to neurocognitive decline. Chronic inflammation, impairment of cerebral microcirculation, and shared risk factors such as hypertension, diabetes, and dyslipidemia contribute to the cognitive decline observed in patients with CVD, highlighting the importance of considering these mechanisms in the integrated management of the disease [4,5,6]. A recent study investigates different aspects of atherosclerosis by analyzing the pathophysiological progression of atherosclerotic lesions, emphasizing the pivotal role of inflammation and its correlations with other diseases, including cognitive decline [7]. Depressive symptoms are approximately twice as common in cardiac patients compared with the general population and are associated with a two- to threefold increased risk of new cardiovascular events within six years [8]. In these patients, depression often presents as apathy and reduced motivation, which significantly impairs treatment adherence. It is not uncommon for patients to express defeatist thoughts, such as “If I’m going to die, why should I make things even harder for myself?” [9]. This outlook can lead to self-destructive behaviors, including the resumption of smoking, alcohol abuse, poor dietary choices, and physical inactivity [10,11]. Conversely, regular physical activity is known to improve both cardiovascular outcomes and depressive symptoms [8].

Anxiety is also frequently observed in CVD patients and is associated with chronic sympathetic activation, elevated cortisol levels, and an increased risk of myocardial infarction and arrhythmias [12]. In some cases, a specific condition known as cardiac anxiety develops, which is characterized by persistent and excessive concern about somatic symptoms perceived as signs of an impending cardiac event. Patients with cardiac anxiety tend to constantly monitor their bodies, frequently seek medical reassurance, and avoid perceived risky situations such as physical exertion [13,14,15,16]. This vicious cycle can exacerbate anxiety and worsen overall quality of life. Nevertheless, both depression and anxiety remain frequently underdiagnosed and untreated in cardiology settings, negatively affecting clinical outcomes and increasing healthcare costs [17,18].

In parallel, cognitive deficits in patients with CVD are emerging as another critical yet often overlooked issue. Recent literature has highlighted a strong connection between cardiovascular and brain health, known as the heart–brain axis [19]. CVDs have been associated with a broad spectrum of cognitive impairments ranging from mild deficits to dementia [19]. Patients with hypertension, atrial fibrillation, and coronary artery disease frequently show impairments in memory, attention, and executive functioning [20,21]. Longitudinal and cross-sectional studies have specifically linked atrial fibrillation to an increased risk of dementia and cognitive decline [22,23,24], while patients with heart failure exhibit significant deficits across multiple cognitive domains [25]. A systematic review and meta-analysis by Wolters et al. [26] found that coronary artery disease increases the risk of severe cognitive impairment by 27%, while heart failure increases it by up to 60%.

Despite these findings, between 50% and 80% of CVD patients with cognitive decline do not receive a formal diagnosis [27], undermining treatment adherence, disease management, and clinical decision-making [28]. This diagnostic gap represents a critical issue in the comprehensive care of cardiac patients.

In light of these considerations, it is clear that anxiety, depression, and cognitive impairment are central elements in the management of CVDs. However, their actual incidence and clinical relevance are often underestimated. This study aims to address this gap by evaluating the presence and impact of these conditions in a sample of patients with CVD and comparing the results with current literature data.

## 2. Materials and Methods

An observational study was conducted to assess the presence of cognitive impairment, depression, and anxiety in people with cardiovascular disease. Seventy-four individuals were recruited from among patients attending the Cardiology Unit of the IRCCS Centro Neurolesi Bonino Pulejo P.O. “Piemonte” in Messina as part of the observational clinical trial titled “Prevention and Treatment of Cardiovascular Disease: Protocol for the Assessment of Psychological, Neuropsychological Implications and Associated Disorders in Cardiovascular Patients” [29], preliminary data of which are reported in this manuscript. The study was approved by the local ethics committee of the IRCCS Centro Neurolesi Bonino Pulejo Neurolesi (Register of opinions: 8/2024) and was subsequently registered on ClinicalTirals.gov (ID: NCT06413823). All subjects provided informed consent.

### 2.1. Population and Setting

A total of 74 subjects (66 M, 8 F; mean age: 66.15 + 8.61 years) were enrolled in the study. Participants were eligible for inclusion if they were between 45 and 85 years old, had a confirmed diagnosis of chronic cardiovascular disease, and provided informed consent.

Exclusion criteria included the presence of severe psychiatric or neurological disorders, oncological diseases, or significant visual impairments that could not be corrected.

Patients were enrolled at the cardiac rehabilitation gym, and the psychological assessments were conducted in person during the rehabilitation process.

Patients had chronic cardiovascular diseases, including previous myocardial infarction, atrial fibrillation, or chronic heart failure. Patients were undergoing cardiac rehabilitation treatment conducted in small groups in the presence of physical therapists, physicians, and nurses. The rehabilitation course was individualized based on their initial health assessment, which included echocardiography, a cardiopulmonary exercise test, and blood tests. In addition, patients were monitored before each rehabilitation session through electrocardiography and the measurement of parameters such as blood pressure and oxygen saturation (SpO_2_), while during each rehabilitation session, the condition of individual patients was constantly monitored through sensors on chest-straps.

### 2.2. Measures

Each participant was assessed through the Montreal Cognitive Assessment (MoCA), Mini-Mental State Examination (MMSE), Beck Depression Inventory-II (BDI-II), and Beck Anxiety Inventory (BAI).

The Montreal Cognitive Assessment (MoCA) is a widely used brief screening tool designed to detect mild cognitive impairment (MCI) in clinical settings around the world [30]. It is also commonly employed in research to assess general cognitive functioning within populations. The MoCA evaluates six key cognitive domains: visuospatial abilities, executive function, attention, language, delayed recall, and orientation. A score of less than 26 out of 30 is the validated threshold for identifying individuals with MCI.

The Mini-Mental State Examination (MMSE) is among the most widely used cognitive screening instruments, designed to assess six cognitive domains: orientation, memory, attention, calculation, language, and visuospatial abilities [31]. It is a validated tool for the early detection of cognitive impairment, with scores below 25 indicating potential deficits.

The Beck Depression Inventory-II (BDI-II) is a 21-item self-report measure used to assess the severity of depressive symptoms over the past two weeks. Each item is rated on a scale from 0 (not at all) to 3 (severely), resulting in a total score ranging from 0 to 63, with higher scores reflecting greater levels of depression [32].

The Beck Anxiety Inventory (BAI) is a 21-item self-report questionnaire designed to assess the severity of anxiety experienced over the past week. Each of the 21 symptoms is rated on a scale ranging from 0 (not present) to 3 (severely present). The total score is calculated by summing the ratings of all individual items [33].

See Table 1 for details of the domains and cutoffs used to configure the various severity levels for individual tests.

### 2.3. Statistical Analysis

Descriptive statistics were used to summarize participants’ sociodemographic characteristics and psychological scores. For categorical variables (e.g., age group, marital status, educational level, employment status, parental status), we calculated absolute frequencies (n) and percentages (%). For continuous variables (e.g., age and MoCA, MMSE, BDI, and BAI scores), we reported means and standard deviations. Independent samples t-tests were performed to compare mean values between male and female participants, using a significance threshold of α = 0.05

### 2.4. Results of Observational Study

Some sociodemographic variables, such as level of education, economic activity, marital status, and having sons, were collected as possible variables that could influence the cognitive and emotional sphere of participants. These variables are summarized in Table 2.

The results indicate that none of the observed differences between males and females were statistically significant at the 0.05 level. This means that, within the context of the current sample, there is no evidence of meaningful gender-based differences in any of the variables analyzed.

Data for individual tests are summarized in Table 3.

In addition, the percentage of occurrence of individual conditions in the whole sample was estimated (Table 4).

Regarding the characteristics of the sample, the overall mean age was 66.15 years, with a standard deviation of 8.61. Female participants had a slightly higher mean age (70.50 years) compared with male participants (65.62 years), although this difference was not statistically significant.

In terms of cognitive function assessment, the mean MoCA score for the entire sample was 22.61 (SD = 4.24), a value indicative of a possible mild cognitive impairment. Male participants had a slightly higher average score (22.80) than female participants (21.00). A similar trend was observed with the MMSE, where the overall mean score was 26.76 (SD = 2.58), falling at the lower end of the normal range. The mean score among men was 26.86, compared with 25.88 among women.

With regard to affective symptoms, the average depression level measured by the BDI-II was 11.09 (SD = 8.74), indicating mild symptomatology. Male participants reported lower scores (10.56) than female participants (15.50). Anxiety levels, measured using the BAI, yielded a mean score of 8.54 (SD = 8.08), consistent with a minimal level of anxiety. Again, women reported lower average scores (5.25) than men (8.94).

Frequency analysis revealed that, according to the MoCA, 32.43% of the sample scored within the normal range, while 35.14% showed mild cognitive impairment and 32.43% showed moderate impairment. In contrast, the MMSE indicated a higher percentage of scores within the normal range (58.11%) compared with the MoCA, while 41.89% showed mild impairment, suggesting that the MMSE may underestimate mild cognitive deficits in comparison with the MoCA.

Regarding depression levels, 56.76% of participants reported minimal symptoms, 21.62% showed mild symptoms, 18.92% showed moderate symptoms, and 2.70% had severe depressive symptoms. A similar distribution was observed for anxiety levels: 58.11% of patients had minimal scores, while 25.68% had mild, 12.16% had moderate, and 4.05% had severe anxiety symptoms.

## 3. Materials and Methods of the Systematic Review

### 3.1. Search Strategy

A systematic review of the literature was conducted to analyze the incidence of cognitive deficits, anxiety, and depression in patients with cardiovascular diseases. The review was carried out following the guidelines of the PRISMA 2020 framework [35]. The literature search was performed across six main databases: PubMed, Web of Science, Cochrane Library, Embase, PsycINFO, and Scopus. All articles published up to 28 March 2025, were included without time restrictions. The following keywords were used:

((cardiovascular disease[Title/Abstract]) OR (myocardial infarction[Title/Abstract]) OR (heart failure[Title/Abstract])) AND ((beck depression inventory) OR (beck anxiety inventory) OR (montreal cognitive assessment) OR (mini mental state examination)).

### 3.2. Inclusion Criteria

A study was considered eligible if it involved adult patients with cardiovascular diseases who underwent cognitive assessment and/or evaluation of depressive and anxiety symptoms using standardized tools. The inclusion criteria were the following: (i) original articles published in English in indexed and peer-reviewed journals; (ii) population consisting of patients with cardiovascular diseases (e.g., heart failure, myocardial infarction, atrial fibrillation, ischemic heart disease, post-cardiac transplant, slow coronary flow); (iii) samples composed of adult subjects aged ≥18 years; (iv) use of at least one of the following validated tools: BDI-II (Beck Depression Inventory-II), BAI (Beck Anxiety Inventory), MoCA (Montreal Cognitive Assessment), or MMSE (Mini-Mental State Examination); (v) observational studies; (vi) no restriction was applied regarding the year of publication.

### 3.3. Exclusion Criteria

Studies that did not include specific data on patients with cardiovascular diseases were excluded. In addition, systematic, integrative, and narrative reviews were discarded. All articles published in languages other than English were also excluded. Studies that did not use MoCA, Mini-Mental, BDI-II, or BAI as assessment tools were excluded in order to ensure a direct and consistent comparison of the data with those analyzed in our study. Patients being treated with psychiatric medications were also excluded to avoid possible interference in the assessment of cognitive function and psychological symptoms. Finally, studies with a design other than observational (e.g., clinical trials, experimental studies, case reports) were excluded.

### 3.4. Study Selection and Quality Assessment

The selection process involved three main phases. First, non-English articles and duplicates were removed. Then, titles and abstracts were screened for relevance and compliance with the inclusion and exclusion criteria. Finally, full-text versions of the remaining studies were reviewed in detail to confirm eligibility and to ensure data completeness.

The quality of the studies included in this review was assessed using the Joanna Briggs Institute’s (JBI) checklists [36].

## 4. Results

**Figure 1:**
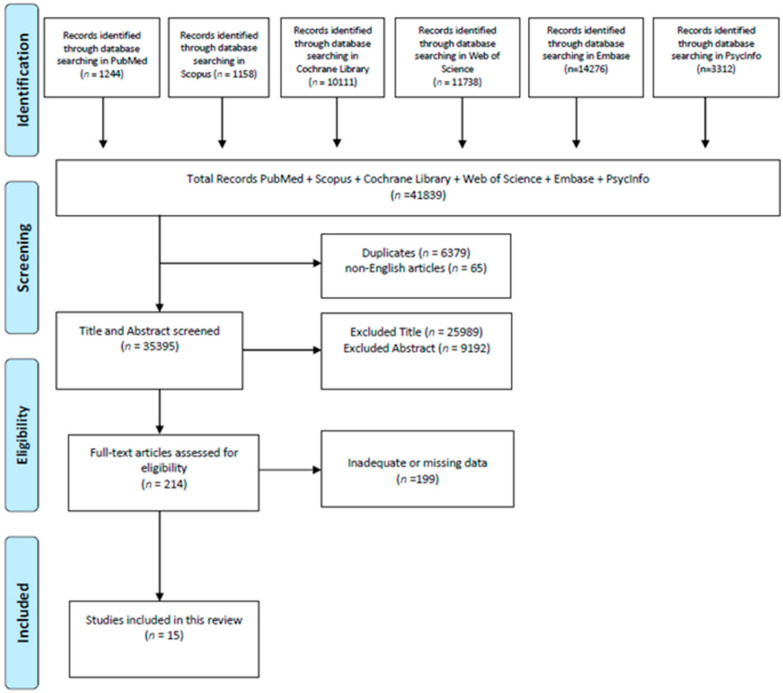
Process of study selection.

The initial search conducted on PubMed, Scopus, Cochrane, Web of Science, Embase, and PsycInfo yielded a total of 41,839 potentially relevant studies. After removing duplicates, titles, abstracts, and full texts were screened to assess relevance and eligibility. Following this selection process, 15 articles met the inclusion criteria and were included in the final analysis (Figure 1).

All studies included in the review investigated the relationship between cardiovascular conditions, cognitive functioning, and depressive and anxiety symptoms (Table 5).

### 4.1. Overall Results

In this review, 15 studies published between 2004 and 2024 were included. Regarding study design, nine were cross-sectional observational studies [37,39,41,42,43,44,45,47,49], four were prospective studies [40,46,48,50], and two were retrospective [38,51].

The studies involved countries from various continents: United States [37,40], United Kingdom [51], Canada [42], Australia [43,50], Sweden and Denmark [38], Ethiopia [39], Pakistan [46], Brazil [41,47], Egypt [45], Serbia [44], Taiwan [49], and the Czech Republic [48].

The number of patients ranged from 33 [47] to 1009 [46], with an average of approximately 210. Procedures varied across studies. In several cases, psychological or neurocognitive tests were administered during hospitalization or outpatient visits [40,41,50]. In other cases, patients completed self-assessment questionnaires independently [46,49].

### 4.2. Cognitive Assessment Results

The Montreal Cognitive Assessment (MoCA) and the Mini-Mental State Examination (MMSE) were used to evaluate cognitive impairment in patients with cardiovascular diseases.

MoCA was employed in five studies [37,38,42,43,50]. The findings highlight a significant prevalence of mild cognitive deficits, although with variations across different clinical settings. For example, Silva et al. [37] found that 23% of elderly patients with heart failure had an MoCA score of below 26, a commonly used threshold indicating the presence of mild cognitive impairment. An even more pronounced result was reported by Blennow Nordström et al. [38], who observed that over 50% of patients who survived out-of-hospital cardiac arrest had a score of <26, suggesting a high incidence of cognitive impairment in this specific population. Similarly, Potter et al. [43] found that 30% of their sample scored below 26, with particularly frequent deficits in executive functions (69%) and delayed recall (93%). In the study by Ball et al. [50], 65% of elderly patients hospitalized for chronic atrial fibrillation had a score of 21, indicative of mild cognitive impairment, with difficulties mainly in the executive, visuospatial, and short-term memory domains. In contrast, Gagnon et al. [42] reported a mean MoCA score of 26.23 in patients with various cardiovascular diseases—a value above the impairment threshold—suggesting a generally preserved cognitive profile in that sample.

The Mini-Mental State Examination (MMSE) was used in two studies [37,44]. In the study by Silva et al. [37], 26% of patients scored below 26, a value indicative of mild cognitive impairment. Similarly, Dikić et al. [44] found that 20.7% of patients post-acute myocardial infarction had a score of below 26, also consistent with mild cognitive impairment.

### 4.3. Depression Assessment Results

The BDI-II was used to assess depressive symptoms in ten studies [39,40,41,44,45,46,47,48,49,51].

Mild depressive symptoms were reported by Trevizan et al. [47], who found that 91% of heart transplant patients had BDI scores of <13. Regarding mild depression, Sherwood et al. [40] found that 29% of patients with heart failure with reduced ejection fraction had a BDI-II ≥14 (overall mean: 10.3). Similar results were observed by De Martini et al. [41], with 26.7% of patients affected;by Kala et al. [48], with a prevalence of 21.5% among post-STEMI patients; and by Kao et al. [49], who reported high values in both men (65%) and women (65.7%). Steeds et al. [51] also found mild symptoms in 47% of post-myocardial infarction patients (BDI ≥12). Additionally, Trevizan et al. [47] reported that 6% of patients had mild symptoms (BDI <19).

Moderate symptoms were identified by Sisay et al. [39], with a mean score of 21.05, and by De Martini et al. [41], who reported a prevalence of 25.6%. Dikić et al. [44] found that 41.5% of their sample had scores of <29, while Husain et al. [46] estimated a mean value of approximately 23.4. Trevizan et al. [47] also reported that 3% of patients had scores consistent with moderate depression (BDI <28). Elamragy et al. [45] noted that most patients with slow coronary flow showed moderate to severe levels of depression, highlighting the psychological severity in this subgroup.

Finally, severe depression was found in a minority of cases, with 7% of patients in the study by De Martini et al. [41]); this was also reported, along with moderate depression, in the study by Elamragy et al. [45].

### 4.4. Anxiety Assessment Results

The Beck Anxiety Inventory (BAI) was used to assess anxiety in three studies [39,45,47]. Minimal anxiety symptoms were observed in the study by Trevizan et al. [47], where 94% of heart transplant patients reported BAI scores of below 7, indicating negligible anxiety symptoms. Only 6% of patients showed mild to moderate anxiety symptoms (BAI < 25), suggesting a low overall incidence of clinically significant anxiety in this population. Moderate anxiety was reported by Sisay et al. [39], who found a mean score of 18.8, consistent with an intermediate level of anxiety symptoms in patients with cardiovascular diseases. Finally, in the study by Elamragy et al. [45], most patients with slow coronary flow had BAI scores of below 26, corresponding to moderate to severe anxiety, highlighting more pronounced psychological distress in this clinical subgroup.

### 4.5. Comparison Between the Present Observational Study with the Literature

Most earlier studies using the MoCA predominantly reported an absence of cognitive impairment cases, often over 70%, with minimal or no moderate CI recorded. In contrast, the present study uniquely shows a near-even split: 32.43% no CI, 35% mild, and 32% moderate. Previous studies using the MMSE have shown higher rates of no CI, ranging from 74% to 79%, with mild CI being a minority. The present study is notable for a lower rate of no CI (58%) and a significantly higher rate of mild CI (42%).

Several previous studies have reported extreme distributions, with some showing 100% of patients with moderate depression or over 90% with no depression, as measured by the BDI-II. The present study reveals a more balanced profile, with 57% with no depression and 22%, 19%, and 3% in the mild, moderate, and severe categories, respectively. Unlike previous studies that favored the extremes, this study captures the full spectrum of depressive symptoms.

Some previous studies have reported 100% with moderate anxiety or over 90% with no anxiety, showing highly skewed distributions. The present study presents a more diverse picture, with 58% with no anxiety and notable percentages of mild (26%), moderate (12%), and severe (4%) anxiety.

**Figure 2:**
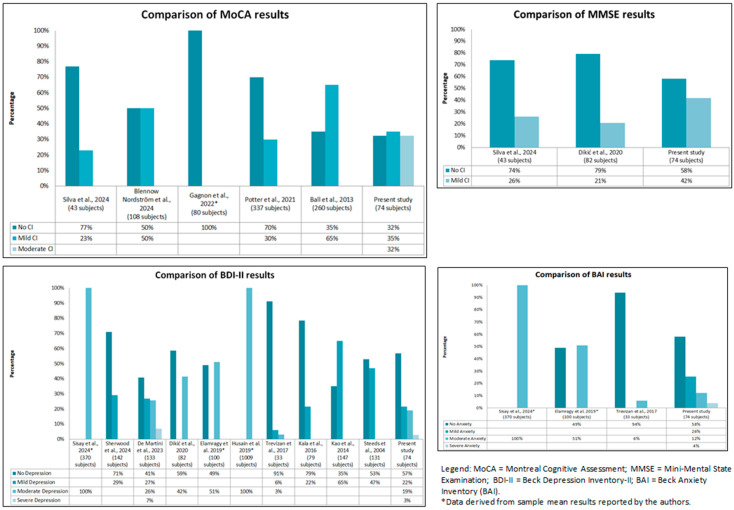
Comparison between the findings of included studies [37,38,39,40,41,42,43,44,45,46,47,48,49,50] and those of the present study.

The comparison between the results of this study and the studies present in the literature for each variable is summarized in Figure 2.

## 5. Discussion

Anxiety, depression, and cognitive impairments are not only threats to mental health but also represent significant risk factors for cardiovascular diseases. Studies [52,53] have shown that these conditions can negatively affect the progression of cardiac disorders by compromising key prognostic aspects, such as treatment adherence, quality of life, frequency of hospitalizations, and long-term mortality. Given their relevance, we considered it essential to analyze the incidence of these disorders in patients with cardiovascular diseases, comparing the collected data with the current evidence available in the literature.

### 5.1. General Results

Our study is observational in nature, and comparisons were planned with other observational studies in the literature that used the same psychological and cognitive tests adopted in this research. This choice ensures methodological consistency between the data collected and the references used, avoiding comparisons with experimental or randomized studies which, due to their different designs, could introduce bias or limitations when compared with our findings.

The comparative analysis revealed that only a few studies employed the Mini-Mental State Examination to assess cognitive impairments, while the majority preferred the MoCA. This preference could be explained by the fact that the MoCA is more sensitive in identifying mild cognitive impairments, making it more suitable in contexts where slight alterations in cognitive function need to be detected [54].

Another interesting finding concerns the evaluation of emotional aspects: depression has been investigated much more frequently than anxiety disorders. This trend is also confirmed by other studies that used instruments other than the BAI and show that anxiety symptoms are still less frequently assessed today [55]. These findings highlight the importance of adopting a more balanced and integrated approach in assessing psychological well-being, recognizing that both depression and anxiety can significantly impact cognitive functioning and quality of life [56]. Although the difference in anxiety scores between men and women did not reach statistical significance, an interesting trend was observed: women reported lower levels of anxiety compared with men in the analyzed sample. This observation, while not conclusive, deserves attention and may reflect gender differences in the way that anxiety is perceived or expressed or the influence of psychological or social factors not assessed in the present study. Further research with larger samples could help clarify the clinical significance of this trend.

### 5.2. Cognitive Impairments

In line with the studies analyzed, our sample exhibited a diverse cognitive profile assessed through the MoCA and MMSE, with a relatively balanced distribution between cognitively normal individuals and those with mild or moderate impairments. Specifically, 32.43% of participants scored within the normal range on the MoCA, while 35.14% and 32.43% showed mild and moderate impairment, respectively. Regarding the MMSE, a higher percentage of individuals fell within the normal range (58.11%), with 41.89% showing mild impairment. These findings are consistent with the literature on cardiovascular patients, although some notable differences emerge.

Several studies [37,38,43,50] report a significant prevalence of mild cognitive impairment, with percentages varying based on the population studied and disease severity. For instance, Silva et al. [37] report that 23% of elderly patients with heart failure had an MoCA score of below 26, a commonly used threshold for detecting mild cognitive decline. In our data, the overall percentage of subjects with mild or moderate cognitive impairment is higher, suggesting either greater sensitivity in our assessment or a sample with different clinical characteristics.

Similarly, Blennow Nordström et al. [38] found that over 50% of patients who survived out-of-hospital cardiac arrest had an MoCA score of <26, reflecting a population at high risk for cognitive dysfunction. Potter et al. [43] also reported a 30% frequency of cognitive impairment, particularly affecting executive function and long-term memory. Our results reveal a comparable distribution, confirming the presence of significant cognitive deficits, especially in executive function and memory domains, as also highlighted by Ball et al. [50]. However, data from Gagnon et al. [42], which report an average MoCA score of 26.23, suggest a generally preserved cognitive profile that contrasts with our sample, where a significant portion presented with moderate impairment. This discrepancy may be due to methodological differences, demographic or clinical characteristics of the samples, or comorbidities that increase the risk of cognitive decline in our cohort.

With regard to the MMSE, the results in our sample (58.11% normal and 41.89% with mild impairment) show a slightly higher prevalence of impairment compared with findings from Silva et al. [37] and Dikić et al. [44], who reported mild cognitive impairment rates of 26% and 20.7%, respectively. This again confirms the lower sensitivity of the MMSE compared with the MoCA, which tends to identify more cases of impairment, particularly those of mild severity.

Further evidence comes from studies using different tools to assess cognitive profiles in patients with cardiovascular diseases. For example, Hamada et al. [57] found that about 50% of a sample of 1,061 patients with heart failure had cognitive decline as measured by the Kihon Checklist, a functional tool widely used in Japanese geriatric settings. This percentage is comparable to the proportion of subjects with mild and moderate impairment in our sample (67.57% on the MoCA), despite methodological and population differences. In the study by Yao et al. [58], 36% of heart failure patients showed cognitive deficits as assessed by the Mini-Cog, a rapid screening test that evaluates memory and orientation. In this case too, the percentage is lower than our MoCA results but closer to those found using the MMSE, confirming how the choice of assessment tool significantly influences the detection of cognitive impairment. Lastly, the study by Park et al. [59], using the CERAD battery, found that patients with cardiovascular diseases scored lower in cognitive tests compared with individuals without such conditions, further confirming the association between cardiac disease and greater cognitive vulnerability.

The results clearly indicate that cognitive decline is a relevant and non-negligible clinical component in these patients, regardless of the test used. The higher sensitivity of the MoCA compared with the MMSE, as well as data from other screening tools like the Kihon Checklist and the CERAD battery, underscore the importance of early and multidimensional cognitive assessment to promptly identify individuals at risk and implement personalized intervention strategies.

Despite the growing evidence of the link between cardiovascular diseases and often-asymptomatic cognitive decline, structured neuropsychological assessment pathways and scheduled personalized rehabilitation interventions, such as cognitive training or neurocognitive stimulation, are still rarely implemented [60]. This lack of intervention limits the ability to detect and address cognitive deficits early, which, if neglected, tend to worsen over time. In light of this, the present study offers a significant contribution by documenting a more balanced distribution of cognitive levels and identifying a higher prevalence of mild impairments compared with other studies. This highlights the need to use more sensitive diagnostic tools such as the MoCA and to maintain strong clinical attention even during the early stages of cognitive deterioration.

### 5.3. Anxiety and Depression

In line with the studies analyzed, the use of the Beck Depression Inventory-II (BDI-II) in our sample revealed a prevalence of depressive symptoms of varying severity: 56.76% of participants showed minimal, 21.62% mild, 18.92% moderate, and 2.70% severe symptoms. These results show a distribution quite similar to that reported in the literature, although with some notable differences. For example, the prevalence of mild depressive symptoms in our sample (21.62%) is lower than that reported by Sherwood et al. [40], who observed that 29% of heart failure patients had BDI-II scores ≥ 14, and by De Martini et al. [41], who reported 26.7%. However, our estimate is comparable to that of Kala et al. [48], who reported a prevalence of 21.5% in patients post-ST-elevation myocardial infarction (STEMI). These findings suggest that mild symptoms are a consistent feature in populations with cardiovascular disease, though their incidence may vary depending on specific clinical and demographic factors.

Regarding moderate depressive symptoms, 18.92% of our sample was affected—a percentage slightly lower than that found by Sisay et al. [39] and De Martini et al. [41], who reported prevalences of 25.6% and average BDI-II scores of around 21.05, respectively. Conversely, Dikić et al. [44] reported a higher percentage of moderate to severe cases, with 41.5% of their population scoring in this range, potentially reflecting differences in patient selection criteria, disease severity, or socio-environmental factors.

The presence of severe depressive symptoms in our sample (2.70%) is lower than that from some reports in the literature, such as those of De Martini et al. [41] and Elamragy et al. [45], where severe depression was documented at higher rates, particularly in subgroups such as patients with slow coronary flow, suggesting a possible link between cardiovascular disease severity and depressive symptom intensity.

To support a broader interpretation, it is helpful to consider studies using tools other than the BDI-II, such as the Patient Health Questionnaire-9 (PHQ-9), which is widely used in clinical practice. For instance, Bahall et al. [61], in a study on hospitalized cardiology patients, found that 78.4% of participants reported non-minimal depressive symptoms and 40% had clinically significant depression (PHQ-9 ≥ 10). Compared with our findings, which indicate an overall prevalence of 43.24% of at least mild symptoms (BDI-II > 13), Bahall’s study shows a higher incidence, likely due to the PHQ-9′s greater sensitivity and the acute hospital setting, which may exacerbate depressive symptoms.

Similarly, Pan et al. [62], in a study of 582 hospitalized patients using the PHQ-9, identified mild depressive symptoms in 21.0% of cases and moderate to severe symptoms in 7.5% during hospitalization. Again, the prevalence of mild symptoms is similar to ours (21.62%), while the proportion with moderate to severe symptoms is lower compared with our combined rate (21.62%), suggesting that, despite using different tools, the detection of severity levels may be comparable, at least for milder forms.

In summary, our study’s results align with the general picture described in the literature, confirming that depressive symptoms are common among patients with cardiovascular disease, with a predominance of mild to moderate forms. The differences observed compared with other studies highlight the importance of considering the clinical context and specific characteristics of the reference population. This underscores the need to use sensitive tools such as the BDI-II for systematic assessment of depression in order to promptly identify at-risk patients and improve overall clinical management.

In line with the reviewed studies, anxiety in our sample, as assessed via the Beck Anxiety Inventory (BAI), showed a predominance of mild symptoms: 58.11% of participants were classified as “minimal,” while 25.68% had mild, 12.16% moderate, and 4.05% severe symptoms. These data suggest a moderate distribution of anxiety within the cardiac population examined, with a significant portion of patients experiencing at least mild or greater symptoms.

Comparison with the literature reveals that anxiety, while a clinically significant component in patients with cardiovascular disease, has been less extensively evaluated than depression. For example, Trevizan et al. [47] found that 94% of heart transplant patients had BAI scores of below 7, indicating negligible anxiety, with only 6% showing mild to moderate symptoms. Similarly, Sisay et al. [39] reported a mean score of 18.8, corresponding to an intermediate level of anxiety in patients with cardiovascular disease, while Elamragy et al. [45] observed that most patients with slow coronary flow had BAI scores of below 26, indicative of moderate to severe anxiety, highlighting greater psychological distress in this subgroup.

Compared to our data, the prevalence of minimal anxiety symptoms is lower (58.11% vs. 94% in Trevizan et al. [47], while the proportion of patients with mild to severe anxiety is higher, suggesting a greater presence of anxious distress in our sample or a different clinical and demographic composition.

For a broader interpretation, it is useful to consider studies using other tools besides the Beck Anxiety Inventory (BAI) to assess anxiety in cardiovascular patients. For example, Williams et al. [63], in a study of 148 patients undergoing cardiac surgery, used the HADS-A (Hospital Anxiety and Depression Scale–Anxiety subscale) and found clinically significant preoperative anxiety in 7% of patients. This prevalence is considerably lower than in our sample, where symptoms above minimal levels (mild, moderate, or severe) were observed in 42% of patients, possibly reflecting a different clinical composition or greater emotional vulnerability in our population, potentially linked to the chronic nature of their cardiac conditions.

Another example comes from the study by Gorini et al. [64], which assessed 2515 cardiac patients using the GAD-7 (Generalized Anxiety Disorder Scale). In this case, the prevalence of significant anxiety was 16%, a figure lower than our overall rate of at least mild anxiety (42%). It is important to note that the GAD-7 focuses specifically on generalized anxiety, while the BAI includes many somatic items that may overlap with typical physical symptoms in cardiac patients (e.g., palpitations, shortness of breath, tremors), potentially inflating scores.

Further data come from a recent meta-analysis by Karami et al. [65], which analyzed 26 international studies and reported a global anxiety prevalence in cardiovascular patients of 32.9%. This value, intermediate between the findings of Williams and Gorini [63,64] and our own, reinforces the clinical relevance of anxiety in cardiology while highlighting significant methodological heterogeneity due to the use of different assessment tools.

Previous studies consistently recognize anxiety as a clinically significant issue in patients with cardiovascular diseases, with potential negative effects on disease progression and patient quality of life. Our findings confirm the importance of anxiety as a relevant psychopathological factor in this context, highlighting the need for increased clinical and research attention. Integrating specific tools, such as the Beck Anxiety Inventory (BAI), into routine assessments could facilitate early recognition and targeted treatment of anxiety, thereby improving overall management of this patient population.

Furthermore, the study highlights a clear association between cognitive dysfunction, anxiety, and depression in patients with cardiovascular diseases. However, it is crucial to consider that mental deterioration is not solely dependent on cardiovascular conditions but is influenced by multiple factors, including family and social support, socioeconomic background, education level, and physical activity. For instance, a higher level of education may promote effective coping strategies, while regular physical activity is associated with better cognitive performance and a lower incidence of mood disorders. Recognizing these factors is essential for accurately interpreting the data and designing personalized and more effective therapeutic interventions.

In light of this evidence, it is imperative to emphasize the importance of early identification and appropriate treatment of anxiety and depression in patients with cardiovascular diseases. Even mild symptoms, if neglected, can significantly compromise quality of life, reduce treatment adherence, hinder physical recovery, and increase the risk of adverse clinical events. Therefore, an integrated and multidisciplinary approach that addresses both psychological and cognitive aspects represents a key strategy for improving clinical outcomes in this population.

Numerous studies highlight the effectiveness of psychological interventions in this context. For example, Fernandes et al. [66] showed that a brief psychological intervention in patients with acute coronary syndrome significantly reduced anxiety and depression levels, in addition to improving illness-related cognition. These benefits were maintained, or even enhanced, at one- and two-month follow-ups, while the control group experienced worsening psychosocial adjustment.

In another study by Cully et al. [67], a six-week cognitive–behavioral therapy (CBT) program in 23 patients with depressive or anxious symptoms and a history of heart failure led to significant improvements, particularly in heart-failure-related quality of life, and moderate reductions in depressive and anxious symptoms.

Finally, a meta-analysis by Li et al. [68], which included 22 studies on patients with coronary artery disease, demonstrated the effectiveness of individualized CBT in reducing depression, anxiety, and stress. Moreover, these interventions had a positive impact on both physical and mental aspects of quality of life.

These findings support the systematic integration of psychological interventions into the care pathways for patients with cardiovascular disease. Such interventions, especially when initiated early, can significantly improve clinical outcomes, psychological well-being, and overall quality of life.

### 5.4. Strengths and Limitations

Our study presents several strengths. Notably, to the best of our knowledge, this is the first study to compare results obtained using the same psychometric assessment tools with those available in the literature, thus offering a direct comparison with pre-existing evidence. This approach helps to fill an important knowledge gap in the field. Furthermore, the integrated approach adopted, which simultaneously considers mental state and cognitive function within the context of cardiovascular disease, represents an additional strength. This perspective promotes a more comprehensive and patient-centered view, reflecting the complexity of the mind–heart interaction in patients with chronic conditions. Exploring these aspects is essential not only to improve the understanding of underlying mechanisms but also to develop more effective and personalized interventions in clinical management.

However, this study also presents some relevant limitations. First, the observational design does not allow for establishing causal relationships between the variables investigated, limiting the ability to draw definitive conclusions about the link between cognitive function and cardiovascular disease. Moreover, the inclusion of various cardiovascular conditions without a systematic distinction between individual pathologies may have introduced a high degree of heterogeneity in the data, affecting the specificity of the results. Added to these limitations is the fact that this is a single-center study with a limited number of patients.

An additional important limitation concerns the sample composition, particularly the extremely small number of female participants (only eight women). However, this reflects a historically recorded finding regarding women’s participation in cardiac rehabilitation, which is estimated at around 11% [69]. This imbalance limits the generalizability of the results to the female population and may have affected the statistical power to detect significant gender differences. Therefore, data relating to sex should be interpreted with caution, and future studies including larger and more balanced samples are needed to further investigate potential gender differences in the phenomena under study. Finally, although it is a strength, the comparison with literature is complicated by methodological variability among the available studies in terms of the populations examined and the types of pathologies considered.

### 5.5. Future Perspectives

The study’s results clearly indicate that the systematic integration of structured neuropsychological assessments into routine cardiology practice is not only desirable but represents an emerging clinical necessity. Ongoing multidimensional monitoring of cognitive functions and emotional states, combined with personalized interventions, such as cognitive stimulation programs and psychological support, could significantly contribute to improving quality of life, treatment adherence, and clinical outcomes.

Future research should involve longitudinal and multicenter designs with the aim of validating integrated intervention protocols and evaluating their effectiveness not only in psychological and cognitive terms but also in regard to long-term cardiovascular outcomes. It is also crucial to promote the implementation of an integrated bio-psycho-social care model within cardiology departments, where psychologists and physicians collaborate synergistically from assessment through to treatment. This would enhance the patient experience, reduce hospital stays, and help contain healthcare costs.

Another promising area for development is the use of telemedicine, which offers effective tools for remotely monitoring patients’ cognitive and emotional conditions, as well as for delivering psychological and rehabilitative interventions. The integration of digital platforms into post-discharge follow-up allows for continuity of care, reducing disparities in access, especially in geographically disadvantaged areas or for patients with limited mobility [70]. Telemedicine can also facilitate the active involvement of patients and caregivers, promoting self-management and greater responsibility for one’s own health.

Lastly, providing support and training for family members or caregivers can significantly improve the management of daily care, reduce the emotional burden, and prevent stress-related situations. Including caregivers in the care pathway strengthens the patient’s support network, increasing the likelihood of a favorable clinical course with positive effects on both quality of life and reduced rehospitalization rates.

## 6. Conclusions

This study confirms the strong interconnection between cardiovascular diseases, cognitive impairments, and psychological disorders such as anxiety and depression, highlighting that these conditions represent not only frequent comorbidities but also significant prognostic factors capable of influencing clinical management and long-term outcomes. The findings reveal a substantial prevalence of mild to moderate cognitive impairment, particularly when assessed using the MoCA, which has proven to be more sensitive than the MMSE in detecting subtle cognitive alterations. The presence of cognitive deficits aligns with existing literature, although in some cases, higher percentages were observed, suggesting either a greater neurocognitive vulnerability in the sample examined or a more effective detection capability of the tool used.

On the psychological front, both depressive and anxious symptoms emerged with notable frequency, with a clear predominance of mild to moderate forms. Depression appears to be more extensively studied in the literature compared with anxiety, but our data emphasize the importance of systematically assessing both dimensions due to their potential impact on quality of life, treatment adherence, and the progression of cardiovascular disease.

Overall, the results suggest the need for a multidimensional approach in the care of cardiology patients, which includes early assessment of cognitive function and the psychological state using standardized and sensitive tools. Timely identification of at-risk individuals would allow for the implementation of targeted interventions, which, despite their proven potential effectiveness, remain underutilized in current clinical practice.

Ultimately, our study reinforces the urgency of integrating neuropsychological and psycho-emotional assessments into the care pathways for patients with cardiovascular diseases in order to ensure a more comprehensive, personalized, and effective management approach.

## Figures and Tables

**Table 1:** Domains and cutoffs of tests used in this observational study.

Test	Domains	Cutoff	Interpretation	References
Montreal Cognitive Assessment (MoCA)	Visuospatial abilities, executive function, attention, language, memory (delayed recall), orientation	26–30: Normal	No cognitive impairment	[30]
		21–25: Mild	Possible MCI	
		11–20: Moderate	Moderate dementia stages	
		0–10: Severe	Severe dementia stages	
Mini-Mental State Examination (MMSE)	Orientation, memory, attention, calculation, language, visuospatial skills	27–30: Normal	No cognitive impairment	[34]
		21–26: Mild	Possible MCI or early dementia	
		11–20: Moderate	Moderate dementia stages	
		0–10: Severe	Severe dementia stages	
Beck Depression Inventory-II (BDI-II)	Depressive symptoms	0–13: Minimal	No clinically significant depression.	[32]
		14–19: Mild	Mild or transient depressive symptoms.	
		20–28: Moderate	Presence of overt depressive symptoms, possible clinical depression.	
		29–63: Severe	Severe symptoms indicative of major clinical depression.	
Beck Anxiety Inventory (BAI)	Anxiety symptoms	0–7: Minimal	Very mild or absent anxiety.	[33]
		8–15: Mild	Mild anxiety symptoms, often consistent with stressful situations.	
		16–25: Moderate	Significant anxiety, which can affect quality of life.	
		26–63: Severe	Intense anxiety, possible clinical anxiety disorder.	

**Table 2:** Sociodemographic variables of participants in the present observational study.

Sociodemographic Variables		n (%)
Gender		
	Male	66 (89.19%)
	Female	8 (10.81%)
Age		
	From 45 to 50 years old	1 (1.35%)
	From 51 to 60 years old	21 (28.38%)
	From 61 to 70 years old	29 (39.19%)
	From 71 to 80 years old	20 (27.03%)
	From 81 to 85 years old	3 (4.05%)
Marital Status		
	Single	10 (13.51%)
	Married	42 (56.76%)
	Widowed	8 (10.81%)
	Divorced	14 (18.92%)
Sons		
	Yes	58 (78.38%)
	No	16 (21.62%)
Educational level		
	Primary education (5 years)	5 (6.76%)
	Lower secondary education (3 years)	16 (21.62%)
	Upper secondary education (5 years)	33 (44.59%)
	Bachelor’s degree	12 (16.22%)
	Master’s degree	7 (9.46%)
	Post-graduate specialization	1 (1.35%)
Economic activity		
	Unemployed	8 (10.81%)
	Working	41 (55.41%)
	Retired	25 (33.78%)

**Table 3:** Participants’ age, sex, and MoCA, MMSE, BDI, and BAI scores.

Variable	Whole Sample Mean (SD)	Male Subjects Mean (SD)	Female Subjects Mean (SD)	*p*-Value
Age	66.15 (8.61)	65.62 (8.38)	70.50 9.91	0.217
MoCA score	22.61 (4.24)	22.80 (4.23)	21.00 (4.24)	0.287
MMSE score	26.76 (2.58)	26.86 (2.57)	25.88 (2.70)	0.356
BDI score	11.09 (8.74)	10.56 (8.17)	15.50 (12.31)	0.302
BAI score	8.54 (8.08)	8.94 (8.27)	5.25 (5.63)	0.127

**Table 4:** Percentage of occurrence of individual conditions in the examined sample.

Test		%
**MoCA**		
	Normal	32.43%
	Mild	35.14%
	Moderate	32.43%
**MMSE**		
	Normal	58.11%
	Mild	41.89%
**BDI**		
	Minimal	56.76%
	Mild	21.62%
	Moderate	18.92%
	Severe	2.70%
**BAI**		
	Minimal	58.11%
	Mild	25.68%
	Moderate	12.16%
	Severe	4.05%

**Table 5:** Characteristics of the included studies.

Study	Aim	Country	Sample	Study Design	Procedure	Measure	Outcomes	Limitations
[37]	To compare the MMSE and MoCA tests for the identification of CD in elderly patients with HF.	United States	43 patients	Cross-sectional observational study	Neuropsychological assessment was performed by a psychologist. Initially, the MMSE test was administered, the results of which were later compared with those obtained from the MoCA test.	MMSE; MoCA	The MoCA identified mild CD (<26) in 23% of the sample, while the MMSE identified mild CD in 26% of the sample.	Lack of sample size calculation. Predominance of low educational level among patients evaluated.
[38]	To evaluate the usefulness of clinical assessment tools in neurocognitive screening after OHCA.	Sweden, Denmark, United Kingdom	108 patients	Retrospective cohort study	Neurocognitive screening was performed as part of the 6-month follow-up.	MoCA; SDMT; TSQ; IQCODE-CA	The MoCA identified a cognitive deficit in 50% of the sample, associated with a total score of <26.	Possible false positives due to nonselective diagnostic criteria.
[39]	Analyze the prevalence of depression and anxiety in patients with cardiovascular disease and identify factors associated with these disorders.	Ethiopia	370 patients	Cross-sectional observational study	Standardized tests were administered as part of the 6-month follow-up.	BDI-II; BAI	The mean score of the BAI was 18.8. The mean score of the BDI-II was 21.05.	Lack of data on confounding variables, which might influence mental health. Results show only an association, not a causal relationship.
[40]	To analyze the association between depressive symptoms and the risk of adverse clinical events in patients with HFrEF.	United States	142 patients	Prospective observational study	Participants underwent medical and psychosocial assessments both at the beginning of the study (baseline) and after 6 months of follow-up.	BDI-II; SCHFI	29% of the sample had a BDI-II ≥ 14 at baseline. The mean BDI-II score at baseline was 10.3.	The sample had better health status than other populations with HFrEF, with higher levels of physical activity and better self-care behaviors.
[41]	To analyze the relationship between depressive symptoms, appetite, and QoL in 86 patients hospitalized for heart failure.	Brazil	133 patients	Cross-sectional observational study	Patients were recruited on the ward, directly in their rooms. After signing the informed consent, they filled out the questionnaire.	BDI-II	The mean score of the BDI-II was 16.3. Within the sample, 25.6% had moderate depressive symptoms, 26.7% had mild depressive symptoms, and 7% had severe depressive symptoms.	The study design limited the generalizability of the results to the entire Brazilian population. Hospital recruitment may have influenced levels of worry and depression because of patients’ perceptions of their own health status.
[42]	To compare cognitive performance in patients with different cardiovascular disease profiles.	Canada	80 patients	Cross-sectional observational study	Cognitive function assessment was performed by a qualified psychometrician or neuropsychologist at the baseline visit.	MoCA	The average MoCA score in the different cardiovascular diseases was 26.23.	Relatively small sample size. The study design did not allow for the detection of cognitive changes over time.
[43]	To examine the prevalence and characteristics of MCI and evaluate its associations with LVD, LA, and AF.	Australia	337 patients	Cross-sectional observational study	Participants were recruited through primary care and advertisements. A medical history, clinical examination, and cognitive assessment were performed. The analysis was based on data collected at baseline.	MoCA	Thirty percent of the sample scored <26, compatible with MCI. Executive functions (69%) and delayed recall (93%) were the most frequently abnormal domains.	Absence of longitudinal patterns. Relatively small sample. Failure to use brain MRI.
[44]	To evaluate the impact of reduced ejection fraction and demographic characteristics on the occurrence of cognitive impairment and depression following myocardial infarction.	Serbia	82 patients	Prospective study	Three months after the diagnosis of acute myocardial infarction, a review of cognitive function was performed during a follow-up visit, and patients were examined for the presence of depressive symptoms.	BDI-II; MMSE	A total of 20.7% of the sample scored <26, consistent with mild cognitive problems, and 41.5% scored <29 in the BDI-II, compatible with moderate depressive symptoms.	Reduced sample size. The use of the MMSE, a screening tool with low sensitivity for mild cognitive impairment, is a limitation.
[45]	To investigate the relationship between psychiatric disorders (anxiety/depression) and CSF.	Egypt	100 patients	Cross-sectional observational study	Psychiatric interviews were conducted blind to CAG results to assess the severity of anxiety and depression.	BDI-II; BAI	Most patients had moderate to severe levels of anxiety and depression, with scores of <29 on the BDI and <26 on the BAI.	The study design does not allow a causal relationship to be concluded. No follow-up is planned. Study parameters cannot be correlated with long-term outcomes.
[46]	To assess the prevalence of depression in a large sample of patients with CHF.	Pakistan	1009 patients	Prospective observational study	Patients were recruited from the cardiology department. The questionnaire was administered at baseline and at six months.	BDI-II	The estimated average BDI score was about 23.4.	Lack of data on length of hospital stay, baseline severity of congestive heart failure (CHF), and adherence to drug therapy.
[47]	To assess psychological disorders, quality of life, and coping strategies in postoperative heart transplant patients.	Brazil	33 patients	Cross-sectional observational study	Participants completed the questionnaires in one individual session during follow-up visits (usually semiannual for most transplant patients) or during other healthcare procedures.	BDI-II; BAI; WHOQOL-BREF; Ways of Coping Scale; MINI International Neuropsychiatric Interview	A total of 91% of the sample had minimal depressive symptoms compatible with a BDI score < 13, 6% showed mild symptoms with a BDI < 19, 3% showed moderate symptoms with a BDI < 28. 94% had minimal anxiety symptoms with a BAI < 7. Only 6% had mild to moderate symptoms with a BAI < 25.	The study design does not allow causal relationships to be established among the variables analyzed. The evolution of phenomena over time cannot be assessed, limiting longitudinal inferences. The absence of a control group reduces the robustness of the conclusions
[48]	To assess the symptoms of depression and anxiety in patients with STEMI treated with primary PCI.	Czech Republic	79 patients	Prospective observational study	Tests were administered within 24 hours of pPCI, before discharge, and subsequently at 3, 6, and 12 months	BDI-II SAS	Within 24 hours after the procedure, 21.5% had symptoms of mild depression (BDI-II ≥ 14).	Lack of data before the onset of MI.
[49]	To determine whether the prevalence of depression in patients with HF differed by gender.	Taiwan	147 patients	Cross-sectional observational study	Participants were recruited at the hospital, provided written informed consent, and individually completed self-assessment questionnaires	BDI-II	Within the sample, 65% of the males and 65.7% of the females had mild depressive symptoms, with a BDI-II score ≥14.	The cross-sectional design allows for the identification of associations but not causality. The relatively small female sample reduced the statistical power, which excluded potential associations with other variables.
[50]	To examine cognitive function in older hospitalized patients with chronic AF.	Australia	260 patients	Prospective observational study	Cognitive function was assessed during hospitalization using the MoCA.	MoCA	Sixty-five percent of the sample had an MoCA score of 21. Multiple deficits were identified in cognitive domains, particularly in executive functions, visuospatial skills, and short-term memory.	Exclusive use of MoCA for cognitive assessment. Potential selection bias due to exclusion of non-English-speaking patients and the influence of an acute clinical setting.

Legend: CD: cognitive deficit; MMSE: Mini-Mental State Examination; MoCA: Montreal Cognitive Assessment; MCI: mild cognitive impairment; HF: heart failure; OHCA: out-of-hospital cardiac arrest; SDMT: Symbol Digit Modalities Test; TSQ: Two Simple Questions; IQCODE-CA: Informant Questionnaire on Cognitive Decline in the Elderly—Cardiac Arrest; BDI-II: Beck Depression Inventory-II; BAI: Beck Anxiety Inventory; MCI: mild cognitive impairment; LVD: left ventricular dysfunction; LA: dysfunction of the left atrium; AF: atrial fibrillation; CSF: slow coronary flow; CAG: coronary angiography; STEMI: ST-Elevation Myocardial Infarction; PCI: Percutaneous Coronary Intervention; SAS: Self-Rating Anxiety Scale; WHOQOL-BREF: World Health Organization Quality of Life-BREF; CHF: congestive heart failure; HFrEF: heart failure with reduced ejection fraction; SCHFI: Self-Care Index of Heart Failure; Qol: QoL: quality of life; CHF: congestive heart failure.

## Data Availability

The data that support the findings of this study are not openly available due to reasons of sensitivity and are available from the corresponding author upon reasonable request.
